# A Nonbinary LDPC-Coded Probabilistic Shaping Scheme for a Rayleigh Fading Channel

**DOI:** 10.3390/e24111649

**Published:** 2022-11-14

**Authors:** Weimin Kang

**Affiliations:** School of Information and Communication Engineering, North University of China, Taiyuan 030051, China; kwm2013@126.com

**Keywords:** probabilistic shaping, nonbinary LDPC, Rayleigh fading

## Abstract

In this paper, a novel, nonbinary (NB) LDPC-coded probabilistic shaping (PS) scheme for a Rayleigh fading channel is proposed. For the NB LDPC-coded PS scheme in Rayleigh fading channel, the rotation angle of 16 quadrature amplitude modulation (QAM) constellations, 64QAM constellations and 256QAM constellations are optimized by the exhaustive search. The simulation results verify the information–theoretical analysis. Compared with the binary LDPC-coded PS scheme for Rayleigh fading channel, the proposed NB LDPC-coded PS scheme can improve error performance. In summary, the proposed NB LDPC-coded PS scheme for Rayleigh fading channel is reliable and thus suitable for future communication systems.

## 1. Introduction

High spectral efficiency (SE) is required in future communication systems, where advanced channel coding schemes and high-order modulation formats play an important role [1,2,3]. In most conventional practical communication systems, when using the uniformly distributed quadrature amplitude modulation (QAM) constellations, it will cause a loss of up to πe/6(≈1.53 dB) toward the Shannon limit [4]. In order to obtain a shaping gain, a probabilistic shaping (PS) scheme was proposed in [5], which utilizes a constant composition distribution matcher (CCDM) [6] to generate different amplitude probability distributions. In [7,8], a binary LDPC-coded rotated QAM-based PS scheme for Rayleigh fading channel was proposed, where a rate 5/6 binary LDPC code was used for both the PS-rotated QAM scheme and the uniform-rotated QAM scheme. The PS scheme would bring rate loss. Therefore, the bit-error-rate (BER) performance, which is examined in [7,8], is not calculated on the basis of SE since the SE changes for different modulation schemes in [7,8]. Therefore, we should evaluate the PS scheme with the same SE and verify the PS gain for the Rayleigh fading channel. For the fading channel, diversity is an efficient method against fading [9]. Constellation rotation can obtain diversity gain for the fading channel, which is widely used for wireless communication standards.

In general, the LDPC codes exhibit better behavior for high code rates when they are compared to turbo codes. In a 5G long code block coding scheme, the LDPC code is used for enhanced mobile broadband service data information. Compared to the binary LDPC codes, the nonbinary (NB) LDPC codes [10] over Galois field GF(*q*) show better error performance for short blocks. $q=2^{p}$, where *p* represents the number of symbolic quantization bits. The NB LDPC-coded PS scheme for an additive white Gaussian noise (AWGN) channel shows excellent error performance in [11,12]. In order to use a flexible LDPC code rate, a NB LDPC-coded hybrid probabilistic shaping scheme was proposed in [13]. However, this work is assumed on an additive white Gaussian noise (AWGN) channel. Considering the wireless fading channel, it is an important issue to evaluate the NB LDPC-coded PS scheme for Rayleigh fading channel and optimize the rotation angle of QAM signals to obtain diversity gain.

In this paper, we propose a NB LDPC-coded PS scheme for Rayleigh fading channel and optimize the rotation angle of QAM signals by the exhaustive search. The theoretical average mutual information (AMI) analysis and simulation error performance show the reliability of the proposed scheme.

The innovation of this paper is shown as follows.

- A NB LDPC-coded two-dimensional PS scheme for Rayleigh fading channel is proposed. Compared with the binary LDPC-coded two-dimensional PS scheme in [7], the proposed NB LDPC-coded two-dimensional PS scheme can obtain coding gain.
- A NB LDPC-coded two-dimensional QAM PS scheme for Rayleigh fading channel based on signal space diversity is proposed. Compared to the NB LDPC-coded two-dimensional QAM PS scheme for Rayleigh fading channel without rotation, the proposed NB LDPC-coded two-dimensional QAM PS scheme for Rayleigh fading channel based on signal space diversity can obtain diversity gain.

The rest of the paper is organized as follows. In Section 2, the system model of the NB LDPC-coded PS scheme for Rayleigh fading channel is proposed. AMI analysis of the proposed PS scheme for Rayleigh fading channel is discussed in Section 3. In Section 4, simulation results are shown. Conclusions are given in Section 5.

## 2. Methods

**Figure 1:**
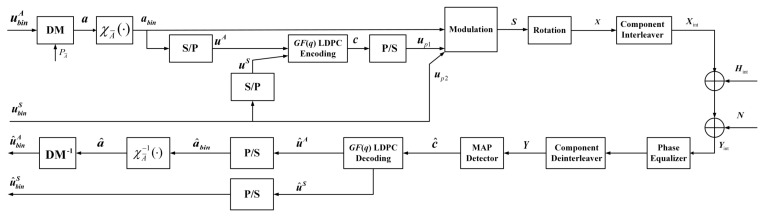
The system model of the proposed NB LDPC-coded probabilistic shaping scheme for Rayleigh fading channel.

The proposed NB LDPC-coded PS scheme for Rayleigh fading channel is shown in Figure 1. In the transmitter, a length of *k* uniform data bits $u_{\mathrm{bin}}^{A}$ is used for generating the nonuniform *n* amplitude symbols according to the unequal probability distribution $P_{\bar{A}}$, which is generated by distribution matcher (DM). For Gray-labeled QAM constellations, such as 16QAM, 64QAM and 256QAM constellations, we consider using two-dimensional PS. Hereby, we use two $\sqrt{M}$-ASK constellations for one M-QAM constellation. Let $\chi_{\bar{A}}\left(\cdot \right)$ denote the amplitude labeling function, where $\chi_{\bar{A}}:\bar{A}\to {\left\{0,1\right\}}^{m-1}$, $m=\frac{\sqrt{M}}{2}$. Assume the binary bits sequence of one 4-ASK symbol is $b_{1}b_{2}$, where $b_{1}$ denotes the sign bit, and $b_{2}$ represents the amplitude bits. The symbols − and + correspond to $b_{1}=0$ and $b_{1}=1$, respectively. The amplitudes 3 and 1 correspond to $b_{2}=0$ and $b_{2}=1$, respectively. Assume the binary bits sequence of one 8-ASK symbol is $b_{1}b_{2}b_{3}$, where $b_{1}$ denotes the sign bit, and $b_{2}b_{3}$ represents the amplitude bits. The symbols − and + correspond to $b_{1}=0$ and $b_{1}=1$, respectively. The amplitudes 7,5,3 and 1 correspond to $b_{2}b_{3}=00$, $b_{2}b_{3}=01$, $b_{2}b_{3}=11$ and $b_{2}b_{3}=10$, respectively. Assume the binary bits sequence of one 16-ASK symbol is $b_{1}b_{2}b_{3}b_{4}$, where $b_{1}$ denotes the sign bit, and $b_{2}b_{3}b_{4}$ represents the amplitude bits. The symbols − and + correspond to $b_{1}=0$ and $b_{1}=1$, respectively. The amplitudes 15, 13, 11, 9, 7, 5, 3 and 1 correspond to $b_{2}b_{3}b_{4}=000$, $b_{2}b_{3}b_{4}=001$, $b_{2}b_{3}b_{4}=011$, $b_{2}b_{3}b_{4}=010$, $b_{2}b_{3}b_{4}=100$, $b_{2}b_{3}b_{4}=101$, $b_{2}b_{3}b_{4}=111$ and $b_{2}b_{3}b_{4}=110$, respectively. For 4-ASK constellations,

(1) $$\chi_{\bar{A}}\left(3\right)=0,\chi_{\bar{A}}\left(1\right)=1.$$

For 8-ASK constellations,

(2) $$\chi_{\bar{A}}\left(7\right)=00,\chi_{\bar{A}}\left(5\right)=01,\chi_{\bar{A}}\left(3\right)=11,\chi_{\bar{A}}\left(1\right)=10.$$

For 16-ASK constellations,

(3) $$\begin{array}{c} \chi_{\bar{A}}\left(15\right)=000,\chi_{\bar{A}}\left(13\right)=001,\chi_{\bar{A}}\left(11\right)=011,\chi_{\bar{A}}\left(9\right)=010,\\ \chi_{\bar{A}}\left(7\right)=100,\chi_{\bar{A}}\left(5\right)=101,\chi_{\bar{A}}\left(3\right)=111,\chi_{\bar{A}}\left(1\right)=110. \end{array}$$

The binary data bits $a_{\mathrm{bin}}$ are converted into $u^{A}$ by series-to-parallel (S/P) conversion, which is one part of the $GF(2^{p})$ LDPC information symbols. We assume that the $GF(2^{p})$ LDPC code rate is *r*, and the length of information symbols and parity symbols are $k_{c}$ and $n_{c}-k_{c}$, respectively. The length of the additional data source bits $u_{\mathrm{bin}}^{S}$ is mnr−(m−1)n. The total code rate is $R=\frac{k+mnr-(m-1)n}{mn}$. $u_{\mathrm{bin}}^{S}$ are converted into $u^{S}$ by S/P conversion, which is the other part of the $GF(2^{p})$ LDPC information symbols. The $GF(2^{p})$ LDPC codes use systematic encoding with the generator matrix G, which can be obtained by the parity-check matrix H, and the parity-check matrix H is constructed by the progressive edge-growth algorithm [14]. After passing through NB LDPC encoding, the uniformly distributed parity symbols c can be transferred into data bits $u_{p1}$ by parallel-to-series (P/S) conversion. The additional data source bits $u_{\mathrm{bin}}^{S}$ are assumed as $u_{p2}$, and the connected data sequences $\{u_{p1}$,$u_{p2}\}$ are used for generating modulated sign parts, while $a_{\mathrm{bin}}$ are used for generating modulated amplitude parts. For one $\sqrt{M}$-ASK constellation symbol, the length of the modulated sign part is one bit, while the length of the modulated sign part is $\frac{\sqrt{M}}{2}-1$ bits. The signal $X={\left[X_{I},X_{Q}\right]}^{T}$ is obtained by the rotation of the traditional signal $S={\left[S_{I},S_{Q}\right]}^{T}$, which can be expressed as

(4) $$\left[\begin{array}{c} X_{I}\\ X_{Q} \end{array}\right]=\left[\begin{array}{cc} \cos\theta & \sin\theta \\ -\sin\theta & \cos\theta \end{array}\right]\left[\begin{array}{c} S_{I}\\ S_{Q} \end{array}\right],$$

where θ is the rotated angle. $X_{I}$ and $X_{Q}$ denote the in-phase signal of *X* and the quadrature signal of *X*, respectively. In order to improve the reliability performance in fading channels, a component interleaver is used, which can make in-phase and quadrature signals go through different fading. Then, the $X_{int}$ signals can be obtained after the component interleaver. Assume that the receiver can get the perfect channel state information; the received signals can be expressed as

(5) $$Y_{int}=H_{int}X_{int}+N,$$

where *N* is the complex Gaussian noise with zero mean and variance $\sigma^{2}$. $H_{int}$ denotes the channel coefficient with zero mean and unit variance.

In the receiver, after passing through the phase equalizer, the received signal is converted into

(6) $$\frac{H_{\mathrm{int}}^{†}}{∥H_{\mathrm{int}}∥}Y_{\mathrm{int}}=∥H_{\mathrm{int}}∥X_{\mathrm{int}}+\frac{H_{\mathrm{int}}^{†}}{∥H_{\mathrm{int}}∥}N.$$

After the component deinterleaver, the signal $Y={\left[Y_{I},Y_{Q}\right]}^{T}$ can be obtained. After maximum a posteriori (MAP) detection, we can get NB LDPC symbol-level log-likelihood ratios. Assume that the label $B=B_{1}B_{2}\cdots B_{p}$ = label$\left(x_{i}\right)$, which means that one $GF(2^{p})$ LDPC code corresponds to one QAM symbol. The received noisy symbol $y_{i}$ is demodulated by calculating $l_{i,j}$,

(7) $$l_{i,j}=\log\frac{P_{B_{j}}\left(0\right)}{P_{B_{j}}\left(1\right)}+\log\frac{q_{i,j}\left(y_{i}|0\right)}{q_{i,j}\left(y_{i}|1\right)},$$

(8) $$P_{B_{j}}\left(b\right)=\sum_{a\in {\left\{0,1\right\}}^{p}:a_{j}=b}P_{B}\left(a\right),$$

(9) $$q_{i,j}\left(y|b\right)=\sum_{a\in {\left\{0,1\right\}}^{p}:a_{j}=b}q_{c}\left(y_{i}|x_{a}\right)\frac{P_{B}\left(a\right)}{P_{B_{j}}\left(b\right)},$$

where $i=1,2,\cdots ,n_{c},j=1,2,\cdots ,p$. $q_{c}\left(\cdot |\cdot \right)$ is a conditional probability density function. $x_{a}=\left\{x_{a_{I}},x_{a_{Q}}\right\}$, $x_{a_{I}}$ and $x_{a_{Q}}$ denote the real part of $x_{a}$ and the image part of $x_{a}$, respectively.

(10) $$q_{c}\left(y|x_{a}\right)=\frac{1}{\sqrt{2\pi \sigma^{2}}}\exp\frac{{-(y_{I}-|h_{I}|\cdot x_{a_{I}})}^{2}-{\left(y_{Q}-|h_{Q}|\cdot x_{a_{Q}}\right)}^{2}}{2\sigma^{2}}.$$

The demodulated bit probability is

(11) $$\begin{array}{c} P\left(y_{i,j}=0\right)=\frac{1}{1+\exp(-l_{i,j})},P\left(y_{i,j}=1\right)=\frac{\exp(-l_{i,j})}{1+\exp(-l_{i,j})}, \end{array}$$

for $i=1,2\cdots n_{c},j=1,2,\cdots p$. The GF$\left(2^{p}\right)$ LDPC decoder input is calculated as

(12) $$P_{i}\left(c\right)=\frac{{\tilde{P}}_{i}\left(c\right)}{\sum_{c^{\prime }\in GF\left(2^{p}\right)}{\tilde{P}}_{i}\left(c^{\prime }\right)},{\tilde{P}}_{i}\left(c\right)=\prod_{j=1}^{p}{\tilde{P}}_{i,j},$$

for $i=1,2\cdots n_{c},j=1,2,\cdots p$, where

(13) $${\tilde{P}}_{i,j}=\left\{\begin{array}{cc} P(y_{i,j}=0), & if{\left[\phi_{GF(2^{p})}^{-1}\left(c\right)\right]}_{j}=0\\ P(y_{i,j}=1), & if{\left[\phi_{GF(2^{p})}^{-1}\left(c\right)\right]}_{j}=1 \end{array}\right.,$$

${\left[\phi_{GF(2^{p})}^{-1}\left(c\right)\right]}_{j}$ is the *j*-th binary mapping bit of *c* for c∈ GF$\left(2^{p}\right)$.

According to [15], the decoding complexity comparison of per bit per iteration for the Log-FFT-BP decoding algorithm of NB LDPC codes and the Log-BP algorithm of binary LDPC codes are shown in Table 1, where $d_{v}$ denotes the average column weight. The regular GF(256) LDPC codes with $d_{v}=2$ and regular GF(2) LDPC codes with $d_{v}=3$ are compared, which shows that the decoding complexity comparison of per bit per iteration for the GF(256) LDPC codes is about 144.7 times as much as that of GF(2) LDPC codes.

The NB LDPC decoding symbols sequence ${\hat{u}}^{A},{\hat{u}}^{S}$ are then mapped via P/S converter. After $\chi_{\bar{A}}^{-1}\left(\cdot \right)$ amplitude inverse labeling function and inverse DM operation, we can finally get the estimated data bits $\left\{{\hat{u}}_{bin}^{A},{\hat{u}}_{bin}^{S}\right\}$.

## 3. Average Mutual Information Analysis

**Figure 2:**
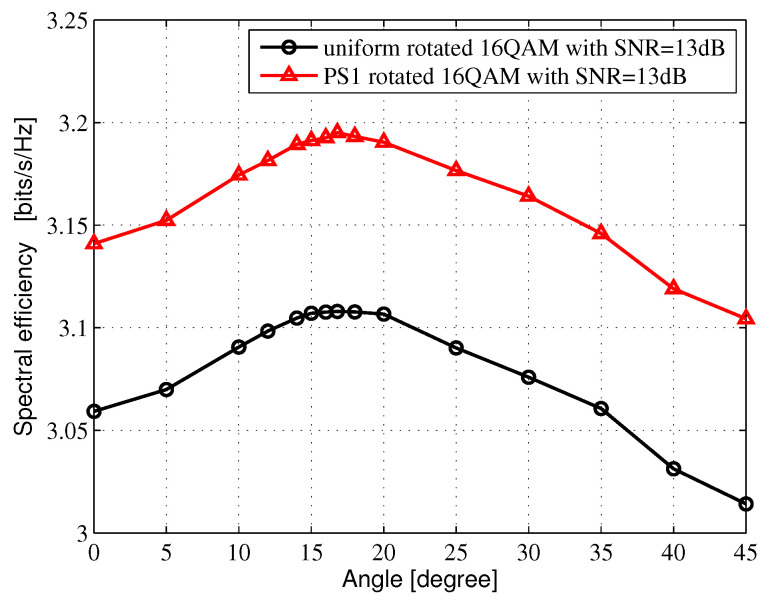
SE vs. θ in 16QAM for Rayleigh fading channel.

**Figure 3:**
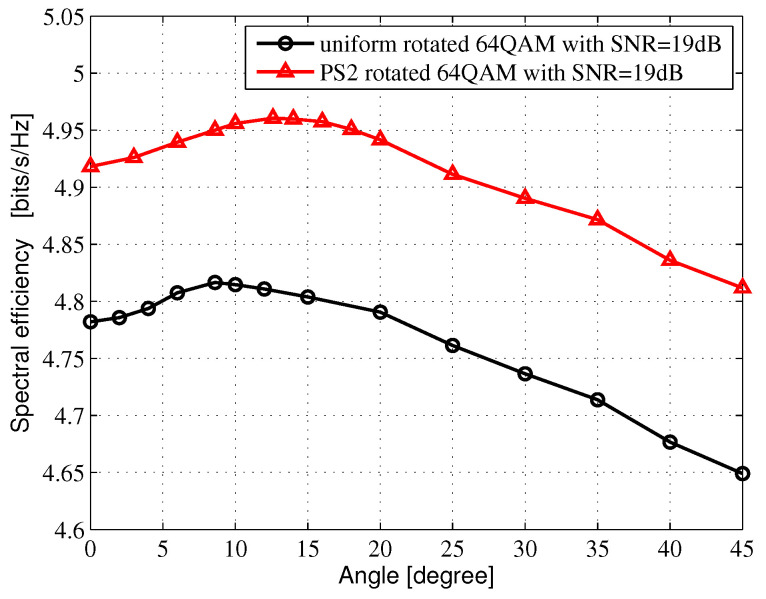
SE vs. θ in 64QAM for Rayleigh fading channel.

**Figure 4:**
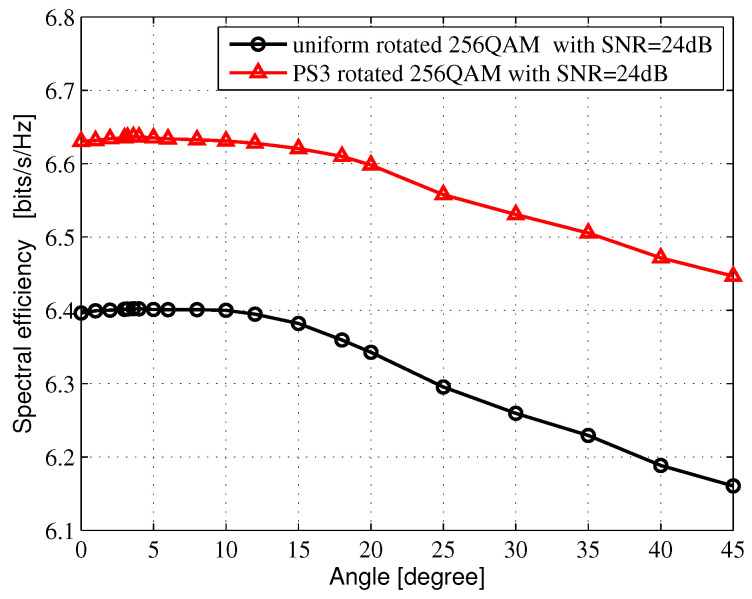
SE vs. θ in 256QAM for Rayleigh fading channel.

The average mutual information (AMI) is the theoretical upper bound, which could be reliably transmitted for given constellation modulation formats. In this section, The regular Nyquist bit-interleaved coded modulation (BICM)-AMI and the PS BICM-AMI for Rayleigh fading channel are investigated. A Gray-labeled *M*-ary QAM constellation consists of two orthogonal $\sqrt{M}$-ASK constellations. Considering the $\sqrt{M}$-ASK constellations, the amplitude probability distribution $P_{X}\left(x\right)$ is selected from Maxwell-Boltzmann (M-B) distribution [7], and $P_{X}\left(x\right)\propto \exp\left(-vx^{2}\right)$, where *x* denotes amplitude. *v* represents the non-negative factor, which can determine the system entropy. When v=0.0, it means that the constellation points are subject to a uniform distribution. When v>0.0, it means that the constellation points are subject to nonuniform distribution. In Figure 2, the amplitude probability distribution for 4-ASK is [3:1]=[0.3505: 0.6495], while the amplitude probability distribution for 8-ASK in Figure 3. is subject to [7:5:3:1]=[0.126429:0.213095:0.301666: 0.358810]. In Figure 4, the amplitude probability distribution for 16-ASK is satisfied to [15:13:11:9:7:5:3:1]=[ 0.0493333: 0.069: 0.0916667:0.116667:0.141333:0.163333:0.18: 0.188667].

The BICM-AMI of the proposed PS system for Rayleigh fading channel can be calculated as

(14) $$\begin{array}{cc} I_{BICM}= & \sum_{i=1}^{m}I\left(c_{i};y|h\right)\\ = & H\left(x\right)-\sum_{i=1}^{m}E_{c_{i},y,h}\left[\log_{2}\frac{\sum_{\bar{x}\in Ø}P\left(y|\bar{x},h\right)P\left(\bar{x}\right)}{\sum_{x\in {Ø}_{c_{i}}}P\left(y|x,h\right)P\left(x\right)}\right], \end{array}$$

where $H\left(x\right)$ represents information entropy, and *h* denotes the fading channel coefficients. χ represents the constellation set. ${Ø}_{c_{i}}$ denotes the constellation subset, where the *i*-th bit $c_{i}\in \left\{0,1\right\}$. $m=log_{2}M$.

We give the objective function of the optimized rotation as follows.

Optimization criterion: Given an SNR, select the optimal rotation angle $\theta^{opt}$ to maximize the SE

(15) $$\theta^{opt}=\underset{\theta \in [0,45]}{\arg\max}\left\{I_{BICM}\left(SNR,\theta \right)\right\}.$$

Figure 2, Figure 3 and Figure 4 show the SE of different rotated angles of θ in 16QAM, 64QAM and 256QAM, respectively, which are obtained from the AMI-optimized criterion by an exhaustive search in Microsoft Visual Studio 2010 software platform. The range of the exhaustive search is from 0 to 45 degrees, and the search interval is set to 0.2 degrees. After passing through the exhaustive search, we can receive the optimal rotated angle $\theta^{opt}$, which can also be obtained through ROC curves by estimating the cutoff points. In Figure 2, when the SNR is 13 dB, the optimal rotated angles $\theta^{opt}$ are both 16.8 degrees for the uniform-rotated 16QAM system and the PS-rotated 16QAM system. In Figure 3, when the SNR is 19 dB, the optimal rotated angles $\theta^{opt}$ are 8.6 and 12.6 degrees for the uniform-rotated 64QAM system and the PS-rotated 64QAM system, respectively. In Figure 2, when the SNR is 24 dB, the optimal rotated angles $\theta^{opt}$ are both 3.6 degrees for the uniform-rotated 256QAM system and the PS-rotated 256QAM system.

**Figure 5:**
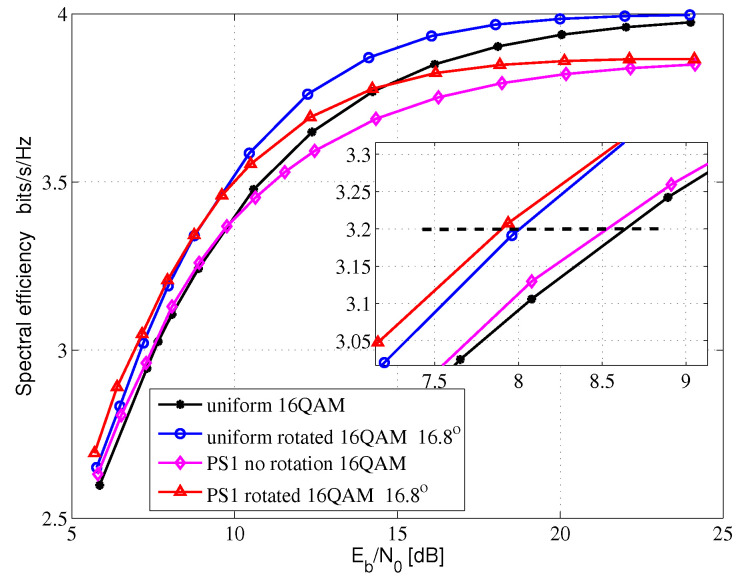
16QAM AMI for Rayleigh fading channel.

**Figure 6:**
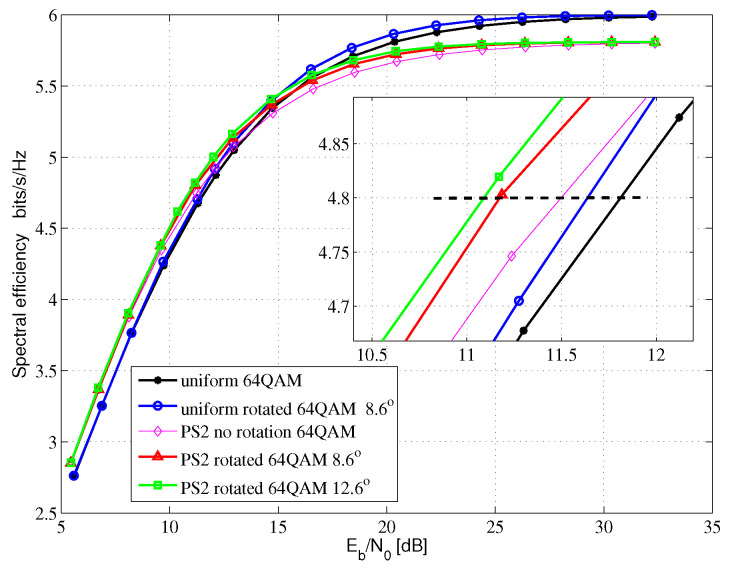
64QAM AMI for Rayleigh fading channel.

**Figure 7:**
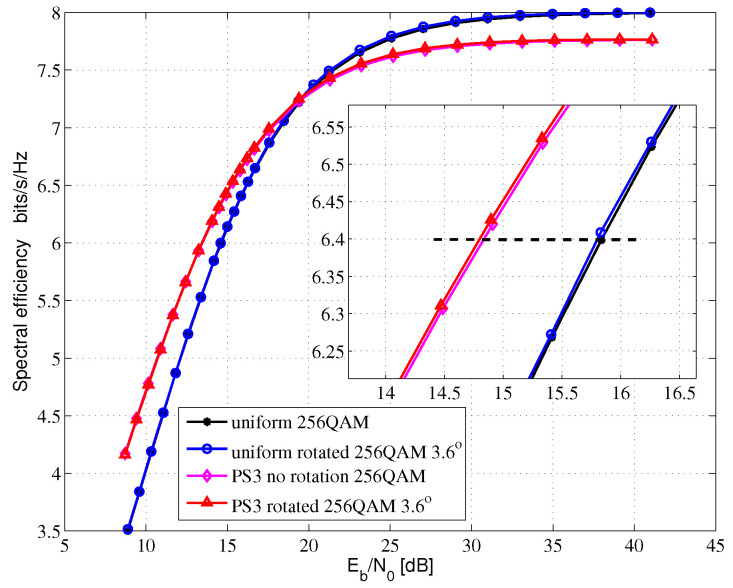
256QAM AMI for Rayleigh fading channel.

Figure 5, Figure 6 and Figure 7 show the AMI comparison among the uniform QAM with no rotation system, the uniform-rotated QAM system, the PS QAM with no rotation system and the PS-rotated QAM system in Rayleigh fading channel for 16QAM, 64QAM and 256QAM, respectively. The rotation angles are 16.8, 8.6 and 3.6 degrees for uniform 16QAM, uniform 64QAM and uniform 256QAM constellations, respectively, which are also used in the DVB-T2 system for fading channel. In this paper, AMI and SE are equivalent.

For the 16QAM system, we compare the SE at 3.2 bits/s/Hz in Figure 5. Compared with the uniform 16QAM with no rotation system, the uniform-rotated 16QAM with a 16.8-degree rotation system, the PS 16QAM with no rotation system and the PS-rotated 16QAM with a 16.8-degree rotation system can obtain 0.65, 0.12 and 0.75 dB theoretical gains, respectively.

For the 64QAM system, we compare the SE at 4.8 bits/s/Hz in Figure 6. Compared with the uniform 64QAM with no rotation system, the uniform-rotated 64QAM with a 8.6-degree rotation system, the PS 64QAM with no rotation system, the PS-rotated 64QAM with a 8.6-degree rotation system and the PS-rotated 64QAM with the optimal 12.6-degree rotation system can obtain 0.18, 0.29, 0.63 and 0.71 dB theoretical gains, respectively.

For the 256QAM system, we compare the SE at 6.4 bits/s/Hz in Figure 7. Compared with the uniform 256QAM with no rotation system, the uniform-rotated 256QAM with a 3.6-degree rotation system, the PS 256QAM with no rotation system and the PS-rotated 256QAM with a 3.6-degree rotation system can obtain 0.04, 1.0 and 1.04 dB theoretical gains, respectively.

## 4. Results

We evaluate the performance gain of the proposed NB LDPC-coded PS scheme in Rayleigh fading channel for 16QAM, 64QAM and 256QAM by Monte-Carlo simulations. The simulated software platform is Microsoft Visual Studio 2010. The frame error rate (FER) performance of the end-to-end communication is considered. The NB LDPC codes over GF(16) have an average variable node degree $d_{v}=2.4$, while the NB LDPC codes over GF(64) and GF(256) have a regular variable node degree of $d_{v}=2$. The LDPC-coded symbol lengths of GF(16), GF(64) and GF(256) are 3000, 2100 and 1500 symbols, respectively. For a fair comparison, the binary LDPC code lengths are 12,000, 12,600 and 12,000 bits in 16QAM, 64QAM and 256QAM, respectively. The binary LDPC codes have a regular variable node degree $d_{v}=3$. The LDPC code rate is set to 4/5 for the uniform QAM with no rotation system and the uniform-rotated QAM system, while the LDPC code rate is set to 5/6 for the PS QAM with no rotation system and the PS-rotated QAM system. The total code rate of the system is set to 4/5. The Log-FFT-BP decoding algorithm is considered for NB LDPC codes, while the Log-BP decoding algorithm is considered for binary LDPC codes. The maximum decoding iteration number of NB LDPC codes and binary LDPC codes are both set to 30. The unequal probabilistic amplitudes yield M-B distributions.

**Figure 8:**
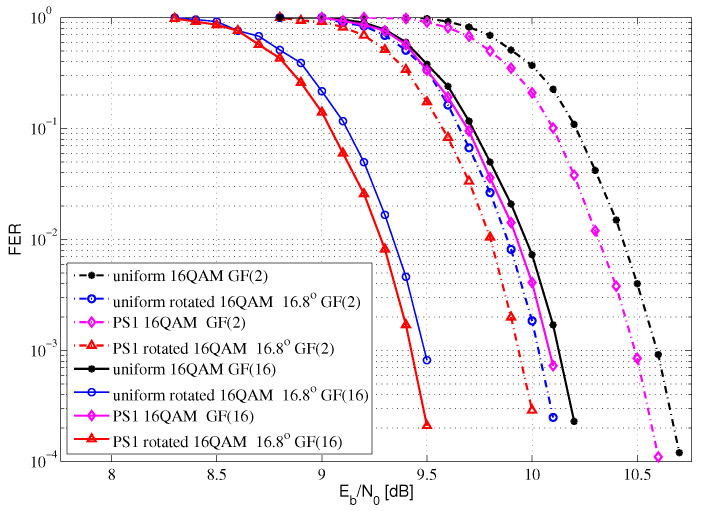
The FER performance of 16QAM for Rayleigh fading channel.

Figure 8 shows the FER performance comparisons of 16QAM for Rayleigh fading channel with SE at 3.2 bits/s/Hz. Compared with the uniform GF(2) LDPC-coded 16QAM with no rotation system, the proposed PS GF(2) LDPC-coded 16QAM with a 16.8-degree rotation system can obtain a 0.65 dB performance gain at FER= $10^{-3}$. Compared with the uniform GF(16) LDPC-coded 16QAM with no rotation system, the proposed PS GF(16) LDPC-coded 16QAM with a 16.8-degree rotation system can obtain a 0.70 dB performance gain at FER= $10^{-3}$. Compared with the uniform GF(2) LDPC-coded 16QAM with no rotation system, the proposed PS GF(16) LDPC-coded 16QAM with a 16.8-degree rotation system can obtain a 1.17 dB performance gain at FER= $10^{-3}$.

**Figure 9:**
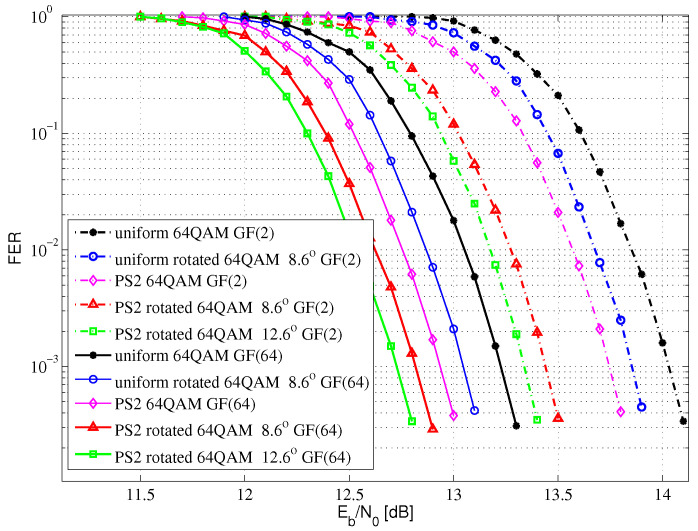
The FER performance of 64QAM for Rayleigh fading channel.

Figure 9 shows the FER performance comparisons of 64QAM for Rayleigh fading channel with SE at 4.8 bits/s/Hz. Compared with the uniform GF(2) LDPC-coded 64QAM with no rotation system, the proposed PS GF(2) LDPC-coded 64QAM with a 8.6-degree rotation system and the proposed PS GF(2) LDPC-coded 64QAM with the optimal 12.6-degree rotation system can obtain 0.60 and 0.70 dB performance gains at FER= $10^{-3}$, respectively. Compared with the uniform GF(64) LDPC-coded 64QAM with no rotation system, the proposed PS GF(64) LDPC-coded 64QAM with the optimal 12.6-degree rotation system can obtain a 0.51 dB performance gain at FER= $10^{-3}$. Compared with the uniform GF(2) LDPC-coded 64QAM with no rotation system, the proposed PS GF(64) LDPC-coded 64QAM with the optimal 12.6-degree rotation system can obtain a 1.31 dB performance gain at FER= $10^{-3}$.

**Figure 10:**
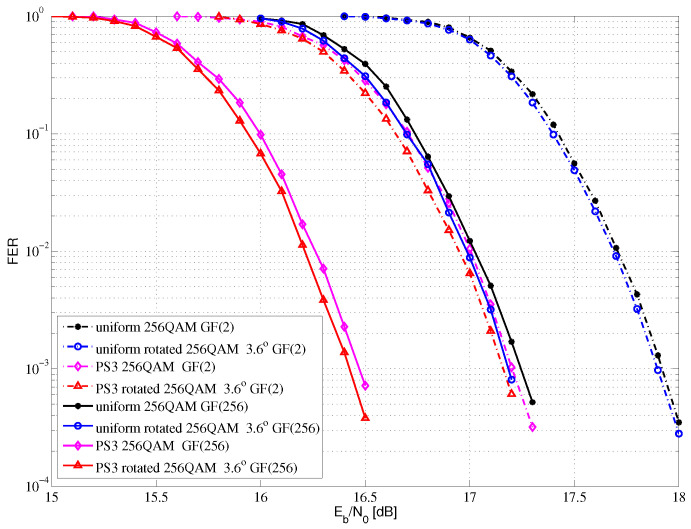
The FER performance of 256QAM for Rayleigh fading channel.

Figure 10 shows the FER performance comparisons of 256QAM for Rayleigh fading channel with SE at 6.4 bits/s/Hz. Compared with the uniform GF(2) LDPC-coded 256QAM with no rotation system, the proposed PS GF(2) LDPC-coded 256QAM with a 3.6-degree rotation system can obtain a 0.77 dB performance gain at FER = $10^{-3}$. Compared with the uniform GF(256) LDPC-coded 256QAM with no rotation system, the proposed PS GF(256) LDPC-coded 256QAM with a 3.6-degree rotation system can obtain a 0.81 dB performance gain at FER = $10^{-3}$. Compared with the uniform GF(2) LDPC-coded 256QAM with no rotation system, the proposed PS GF(256) LDPC-coded 256QAM with a 3.6-degree rotation system can obtain a 1.49 dB performance gain at FER = $10^{-3}$.

In summary, compared with the binary LDPC-coded QAM with no rotation system, the proposed PS nonbinary LDPC-coded QAM with a rotation system can obtain shaping gain, coding gain and diversity gain. In Figure 8, Figure 9 and Figure 10, there are gaps between the groups of curves, which is due to the selected component.

## 5. Conclusions

In this paper, a NB LDPC-coded PS scheme for Rayleigh fading channel is proposed. The simulation results coincide well with the theoretical AMI analysis, which shows that the NB LDPC-coded PS scheme is suitable for Rayleigh fading channel, and the shaping gain can be obtained. In summary, the proposed NB LDPC-coded PS scheme for Rayleigh fading channel is robust and reliable, which is a potential scheme for the future communication system. In the future, we will consider the Rayleigh flat-fading channel with Doppler shift and consider the question of error protection inequality between the bits transmitted as the sign bits and those represented by the amplitude.

## Figures and Tables

**Table 1:** The decoding complexity of Log-FFT-BP and Log-BP per bit per iteration.

Operation	Log-FFT-BP for NB LDPC	Log-BP for Binary LDPC	GF(256) Regular LDPC, $d_{v}=2,p=8$	GF(2) Regular LDPC $d_{v}=3$
addition/subtraction	$\left(2q+\frac{4q}{p}\right)d_{v}$	$4d_{v}-1$	1280	11
comparison	$2qd_{v}$	–	1024	–
table look-up	$2qd_{v}$	$4d_{v}$	1024	12
total	$\left(6q+\frac{4q}{p}\right)d_{v}$	$8d_{v}-1$	3328	23

## Data Availability

Not applicable.
